# Impact of metformin and *Dysosmobacter welbionis* on diet-induced obesity and diabetes: from clinical observation to preclinical intervention

**DOI:** 10.1007/s00125-023-06032-0

**Published:** 2023-10-28

**Authors:** Emilie Moens de Hase, Audrey M. Neyrinck, Julie Rodriguez, Miriam Cnop, Nicolas Paquot, Jean-Paul Thissen, Yining Xu, Ana Beloqui, Laure B. Bindels, Nathalie M. Delzenne, Matthias Van Hul, Patrice D. Cani

**Affiliations:** 1 Metabolism and Nutrition Research Group, Louvain Drug Research Institute (LDRI), UCLouvain (Université catholique de Louvain), Brussels, Belgium; 2 WELBIO-Walloon Excellence in Life Sciences and Biotechnology, WELBIO Department, WEL Research Institute, Wavre, Belgium; 3 ULB Center for Diabetes Research, Université Libre de Bruxelles, and Division of Endocrinology, Erasmus Hospital, Brussels, Belgium; 4 Laboratory of Diabetology, Nutrition and Metabolic Disease, Université de Liège, Liège, Belgium; 5 Pole of Endocrinology, Diabetes and Nutrition, Institute of Experimental and Clinical Research (IREC), UCLouvain (Université catholique de Louvain), Brussels, Belgium; 6 Advanced Drug Delivery and Biomaterials, Louvain Drug Research Institute (LDRI), UCLouvain (Université catholique de Louvain), Brussels, Belgium; 7 Institute of Experimental and Clinical Research (IREC), UCLouvain (Université catholique de Louvain), Brussels, Belgium

**Keywords:** Adipose tissue, *Dysosmobacter welbionis* J115^T^, Glucose tolerance, Gut microbiota, Metformin, Obesity, Prebiotics, Type 2 diabetes

## Abstract

**Aims/hypothesis:**

We aimed to investigate the association between the abundance of *Dysosmobacter welbionis*, a commensal gut bacterium, and metabolic health in human participants with obesity and diabetes, and the influence of metformin treatment and prebiotic intervention.

**Methods:**

Metabolic variables were assessed and faecal samples were collected from 106 participants in a randomised controlled intervention with a prebiotic stratified by metformin treatment (Food4Gut trial). The abundance of *D. welbionis* was measured by quantitative PCR and correlated with metabolic markers. The in vitro effect of metformin on *D. welbionis* growth was evaluated and an in vivo study was performed in mice to investigate the effects of metformin and *D. welbionis* J115^T^ supplementation, either alone or in combination, on metabolic variables.

**Results:**

*D. welbionis* abundance was unaffected by prebiotic treatment but was significantly higher in metformin-treated participants. Responders to prebiotic treatment had higher baseline *D. welbionis* levels than non-responders. *D. welbionis* was negatively correlated with aspartate aminotransferase (AST) and alanine aminotransferase (ALT) levels and fasting blood glucose levels in humans with obesity and type 2 diabetes. In vitro, metformin had no direct effect on *D. welbionis* growth. In mice, *D. welbionis* J115^T^ treatment reduced body weight gain and liver weight, and improved glucose tolerance to a better level than metformin, but did not have synergistic effects with metformin.

**Conclusions/interpretation:**

*D. welbionis* abundance is influenced by metformin treatment and associated with prebiotic response, liver health and glucose metabolism in humans with obesity and diabetes. This study suggests that *D. welbionis* may play a role in metabolic health and warrants further investigation.

**Clinical trial:**

NCT03852069

**Graphical Abstract:**

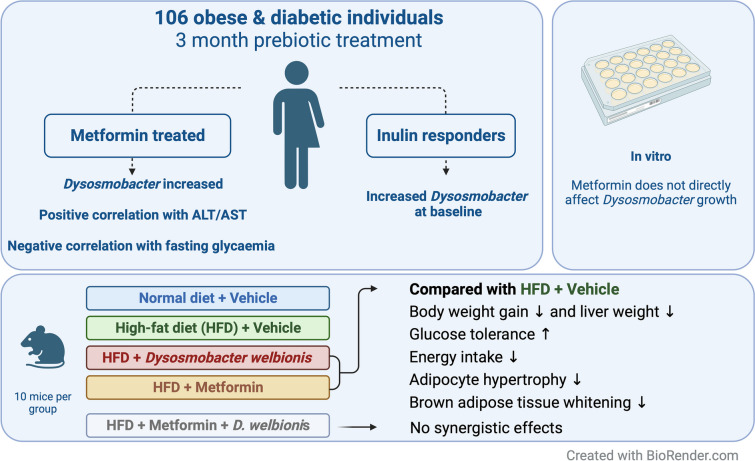

**Supplementary Information:**

The online version contains peer-reviewed but unedited supplementary material available at 10.1007/s00125-023-06032-0.

## Introduction


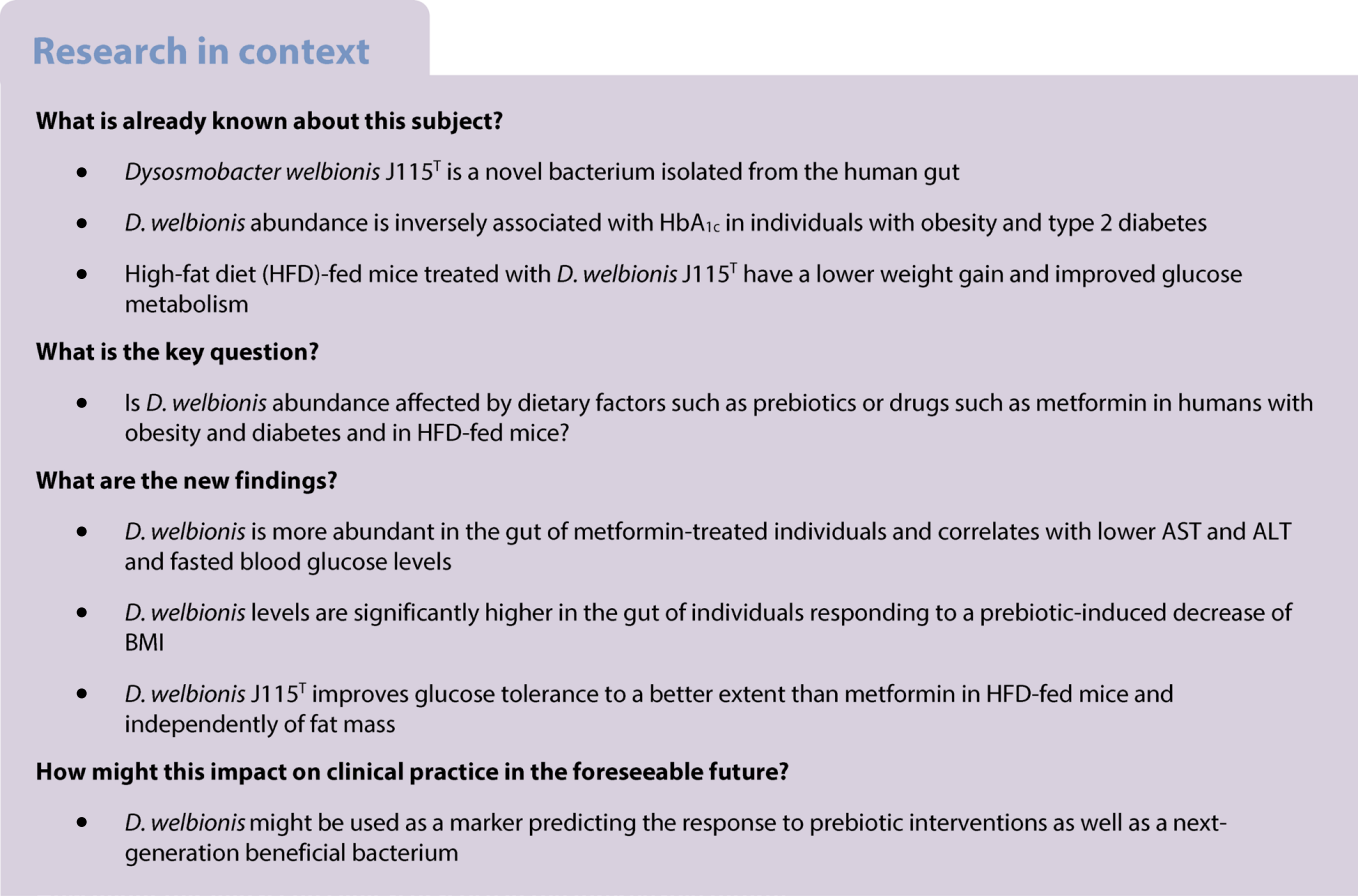


In 2021, 537 million adults were living with type 2 diabetes worldwide. That same year, the IDF reported that, globally, another 541 million adults had impaired glucose tolerance and were therefore at high risk of developing type 2 diabetes [1]. With such alarming numbers, it is crucial to deepen our understanding of the physiopathology underlying this disease and to identify potential novel therapeutic targets. Among the various possible mediators, the gut microbiota is regarded as a pivotal factor influencing host physiology [2, 3]. Many observational studies are revealing various connections between the composition and/or functionality of the gut microbiota and diseases in the host [4]. Nonetheless, establishing a causal relationship for particular taxa remains challenging [5]. In the past 10 years, researchers have identified promising next-generation beneficial bacteria. Among these, the symbiotic bacterium *Akkermansia muciniphila* has undergone extensive investigation in preclinical research and has been recognised as a potential novel approach to address metabolic disorders and associated diseases [6]. Evidence suggests that *A. muciniphila* is directly influenced by different dietary regimens such as specific dietary fibres (e.g. the prebiotic inulin-type fructans) [7–9] and specific drug treatments such as the glucose-lowering drug metformin [10–13]. Inulin-type fructan treatments have been linked to changes in host metabolism (i.e. glucose and lipid metabolism and inflammation), gut microbiota and specific metabolites (including short-chain fatty acids [SCFAs]) [14–17]. We recently identified a novel butyrate-producing bacterium which we named *Dysosmobacter welbionis* J115^T^ [18]. Using various human cohorts representing the general population (totalling about 12,000 individuals), we found that *D. welbionis* was detected in about 70% of the samples screened, making it a prevalent bacterium [19]. For comparison, *A. muciniphila* is detected in 70–80% of the general population when using part of the same cohorts [20]. Using a cohort of participants with obesity and type 2 diabetes, we previously discovered that *D. welbionis* negatively correlated with BMI, fasting glucose and HbA_1c_. We found no direct correlations with BMI and weight in non-diabetic participants, although we showed in preclinical studies that supplementation of *D. welbionis* strain J115^T^ to high-fat diet (HFD)-fed mice reduced body weight and fat mass gain. This supplementation was associated with improved glucose homeostasis and increased non-shivering thermogenesis, coupled to a higher number of mitochondria in the brown adipose tissue (BAT) [19]; however, the molecular mechanisms are still unknown. Whether specific dietary interventions or metformin treatments influence the abundance of *D. welbionis* also remains unknown.

In this study, we combined human intervention studies, in vitro studies and in vivo mouse experiments to (1) investigate whether individuals with obesity receiving high doses of the prebiotic inulin (16 g/d as a supplement, coupled to dietary advice to consume inulin-rich vegetables for 3 months) exhibited specific changes in faecal *D. welbionis* abundance compared with individuals with obesity receiving supplements of maltodextrin, a starch-derived carbohydrate with poor prebiotic capacities, and advised to consume inulin-poor vegetables; (2) examine if metformin-treated individuals have a different abundance of *D. welbionis* in their gut compared with non-treated individuals; and (3) compare the effects of *D. welbionis* J115^T^ supplementation vs metformin administration on glucose intolerance and fat mass in mice on HFD.

## Methods

For detailed methods and human and animal ethics, please refer to the electronic supplementary material (ESM) Methods.

### Human cohort

The Food4Gut study was a 3-month-long, multicentric, single-blind, placebo-controlled trial. Recruitment, enrolment, randomisation, sample size determination, inclusion and exclusion criteria, and outcomes were previously described [16]. Male and female participants (106 individuals) were recruited in three university hospitals in Belgium (Cliniques universitaires Saint-Luc and Hôpital Erasme in Brussels, and Centre Hospitalier Universitaire in Liège) as previously described [14–16]. Details of the inclusion and exclusion criteria and sampling methods are shown in the ESM Methods.

### Quantitative PCR of *D. welbionis* and total bacteria in human samples

Absolute quantification of total bacterial load and *D. welbionis* was performed by quantitative PCR (qPCR) using a QuantStudio3 (Applied Biosystems, the Netherlands) with SYBR Green (GoTaq qPCR mix, Promega, USA) for detection and using the QuantStudio software (version 1.4.3, Applied Biosystems, the Netherlands).

### Culture and preparation of *D. welbionis* for mouse experiments

*D. welbionis* J115^T^ was cultured anaerobically in a modified yeast extract, casitone and fatty acids (YCFA) medium supplemented with 10 g/l inositol. Cells were harvested, immediately frozen in anaerobic vials and stored at −80°C.

### Mouse experiments

#### Mouse model

Sets of 7-week-old C57BL/6J male mice (Janvier Laboratories, Le Genest-Saint-Isle, France) were housed in pairs in SOPF (specific opportunistic and pathogen free) conditions, in a controlled environment with free access to sterile (irradiated) food and sterile (autoclaved) water.

A set of 50 mice was randomly divided into five groups of ten mice (housed two per cages). The mice were fed either control diet (CT) (AIN93Mi) or HFD (D12492, Research Diets, USA), with a daily oral supplementation of *D. welbionis* J115^T^, metformin, *D. welbionis* J115^T^ + metformin or vehicle solution. The treatment continued for 10 weeks. Mice were killed and dissected by experimenters masked to the treatment groups after a 6 h fasting period.

#### OGTT

One week before the end of the experiment, fasted mice were given an oral gavage glucose load. Blood glucose was regularly measured 30 min before to 2 h after oral glucose load. Plasma insulin concentration was also measured.

#### Mouse tissue sampling

Blood samples and tissue samples (liver, BAT, subcutaneous adipose tissue, mesenteric adipose tissue) were dissected and stored at −80°C for further analysis. Part of the adipose tissue was fixed for histological analysis.

#### The insulin resistance index

The insulin resistance index was determined by multiplying the area under the curve (from −30 to 15 min) of blood glucose and plasma insulin obtained during the OGTT.

#### Mouse histological analyses

Brown and white adipose tissues were fixed in 4% paraformaldehyde for 24 h at room temperature. Samples were then processed for paraffin embedding and stained for further analysis using a Panoramic ScanII slide scanner (3DHistech, Hungary) and visualised with the Cytomine web platform (https://cytomine.com, Belgium).

#### Gene expression analysis by real-time qPCR

Real-time PCR was performed with the StepOnePlus real-time PCR system and software (Applied Biosystems, Den Ijssel, the Netherlands) using GoTaq qPCR mix (Promega, USA) for detection, according to the manufacturer’s instructions. Primer sequences for the mouse genes are shown in ESM Table 1.

#### Mouse plasma multiplex analysis

Portal vein plasma levels of total glucagon-like peptide-1 (GLP-1), peptide YY (PYY), ghrelin, leptin and glucagon were measured by multiplex assay kits based on chemiluminescence detection, following the manufacturer’s instructions (Meso Scale Discovery [MSD], Rockville, MD, USA). Analyses were performed using a QuickPlex SQ 120 instrument (MSD) and DISCOVERY WORKBENCH 4.0 software (MSD).

### Animal ethics

All mouse experiments were approved by the Ethical Committee for Animal Care of the Health Sector of the Université catholique de Louvain (UCLouvain) and were performed in accordance with the guidelines of the Local Ethics Committee and in accordance with the Belgian Law of 29 May 2013 regarding the protection of laboratory animals (agreement number LA1230314).

### GLP-1 stimulation on murine L cells (GLUTag cells)

GLUTag cells (donated by D. Drucker [University of Toronto]) were plated on 1% Matrigel-coated 24-well plates. *D. welbionis* J115^T^ cells were suspended in the cell line medium at increasing concentrations and incubated in the presence of dipeptidyl peptidase IV (DPP-IV) inhibitor. Total GLP-1 levels in collected supernatants and cell lysates were measured using an MSD ELISA kit.

### Statistical analyses

Statistical analyses were performed using GraphPad Prism version 9.4.0 (GraphPad Software, Boston, MA, USA, http://www.graphpad.com/) and RStudio program (RStudio 2022.07.1 for MacOS, PBC, Boston, MA, USA, http://www.rstudio.com/). Details about the statistical test used can be found in the figure legends. Data are presented as mean±SEM. Outliers were tested and removed using the ROUT test when *p*<0.01 (Prism 9.4v).

## Results

### *D. welbionis* is not affected by prebiotic treatment but is highly abundant in metformin-treated individuals

**Fig. 1:**
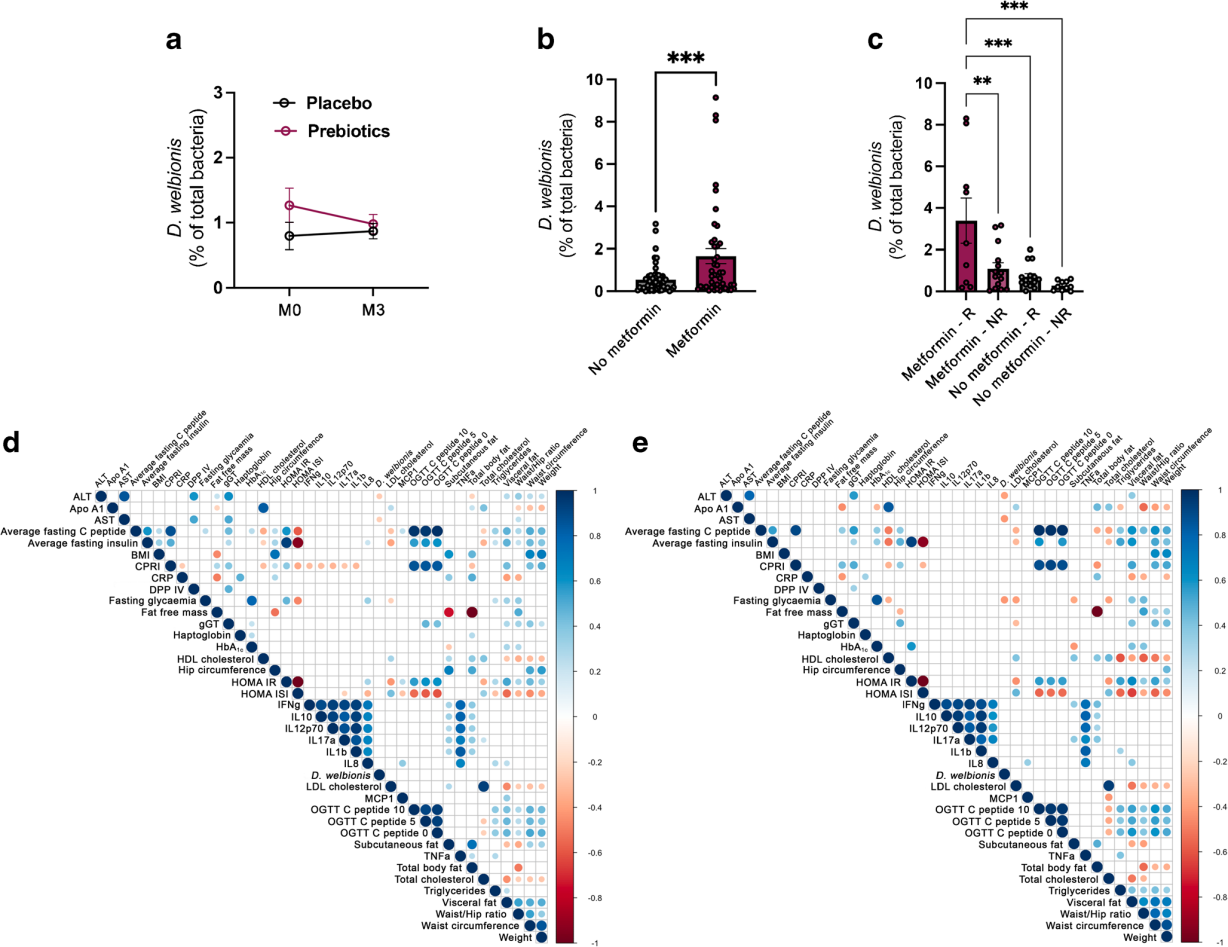
*D. welbionis* levels are not affected by prebiotics but are higher in metformin-treated individuals and responders. (**a**) Faecal *D. welbionis* levels (% of total bacteria) before (M0) and 3 months after (M3) supplementation with prebiotics or a placebo. (**b**) Faecal *D. welbionis* levels (% of total bacteria) at baseline in participants treated with metformin (metformin) or not (no metformin). (**c**) Faecal *D. welbionis* levels (% of total bacteria) at baseline in participants treated or not with metformin and responders (R) or non-responders (NR) to the prebiotic treatment for BMI decrease. (**d**) Correlation analysis between anthropometric and metabolic variables and *D. welbionis* measured in the gut microbiota of all individuals included in the cohort prior intervention (baseline, M0). (**e**) Correlation analysis between anthropometric and metabolic variables and *D. welbionis* measured in the gut microbiota of metformin-treated individuals included in the cohort prior intervention (baseline, M0). Results are presented as bar plots with mean±SEM for parts (**a**), (**b**) and (**c**). Data were analysed using unpaired *t* test for part (**b**) and one-way ANOVA followed by Tukey’s post hoc test for part (**c**). **p<0.01; ***p<0.001. For parts (**d**) and (**e**), the presence of a circle indicates that the correlation is significant, *p*<0.05 (Spearman’s correlation test). The circle size relates to the absolute value of correlation. The bigger the circle size, the higher the correlation. Blue indicates positive correlations and red negative correlations. Apo, apolipoprotein; CPRI, C-peptide to glucose ratio; CRP, C-reactive protein; DPP IV, dipeptidyl peptidase IV; gGT, γ-glutamyl transferase; MCP1, monocyte chemoattractant protein 1

In total, 106 participants (placebo *n*=55, prebiotics *n*=51) were included in the analysis. As previously reported, the groups were similar in terms of age, sex, weight and BMI at baseline [16]. We first aimed to identify whether the abundance of *D. welbionis* was affected by treatment with prebiotics. We found that *D. welbionis* was unaffected by the dietary intervention (Fig. 1a).

Given that metformin has previously been shown to be a confounding factor when assessing gut microbiota composition in type 2 diabetic individuals [10–13], we analysed the baseline data separately in metformin-treated (metformin) and metformin-naive (no metformin) participants [16]. Of 106 individuals, 59 were metformin-naive (prebiotics *n*=27, placebo *n*=32) and 47 were metformin-treated (all diabetic, prebiotics *n*=24, placebo *n*=23). Using this stratification, we discovered that metformin-treated individuals had significantly higher levels of *D. welbionis* in their faeces (Fig. 1b) at baseline, strongly suggesting that the abundance of *D. welbionis* is influenced by this glucose-lowering drug.

### *D. welbionis* at baseline is more abundant in individuals responding to prebiotic treatment

In our previous study, we observed that prebiotic treatment did not result in a reduction of BMI for all individuals, indicating individual variability in the response to this intervention [14]. After 3 months of prebiotic treatment, some participants exhibited a significant decrease of 2 BMI units (responders) whereas others showed no change (non-responders). We formerly linked this observation with a specific composition of the gut microbiota at baseline and were able to confirm a causal role for the microbiota in a mouse model (transplantation of the gut microbiota from individuals with obesity to HFD-fed mice) [14]. However, at the time of these analyses, *D. welbionis* was still unknown. In this study, we investigated whether the abundance of *D. welbionis* could be linked with the status of responders vs non-responders to the prebiotic treatment on BMI decrease. Strikingly, we found that individuals who responded to the prebiotic treatment had almost three times higher faecal levels of *D. welbionis* at baseline. This effect persisted even after stratification of individuals by metformin treatment. Thus, non-responders had significantly lower *D. welbionis* in their gut at baseline, a difference that was even more pronounced when comparing metformin-treated vs non-metformin-treated individuals (Fig. 1c).

### *D. welbionis* is negatively correlated with AST, ALT and fasted blood glucose levels in humans with diabetes and obesity

At baseline, we found that *D. welbionis* was significantly inversely correlated with alanine aminotransferase (ALT) and aspartate aminotransferase (AST) in the entire cohort, but not with any other variables (Fig. 1d). When considering metformin treatment, this correlation was lost in the non-metformin group, whereas in the metformin group *D. welbionis* was not only significantly inversely correlated with ALT and AST, but also inversely correlated with fasted blood glucose levels (Fig. 1e).

### Metformin does not directly affect growth of *D. welbionis* J115^T^ in vitro

Taken together, these observations suggest that *D. welbionis* is strongly influenced by metformin use. Metformin has been shown to alter the composition and metabolic activity of gut microbiota, with some bacteria being able to utilise it as a carbon source and grow in its presence [21]. Therefore, we explored in vitro whether metformin directly influences the growth of *D. welbionis* J115^T^. We found that the growth curves of *D. welbionis* J115^T^ were affected by the presence or absence of its unique carbon source (i.e. inositol [18]) but not by metformin (ESM Fig. 1). These data suggest that the increase in *D. welbionis* observed in human gut is likely explained by a specific interaction in a complex environment and not by the direct utilisation of metformin as an energy source.

### Metformin and *D. welbionis* J115^T^ reduce diet-induced obesity, fat mass accumulation and liver weight without synergistic effects

**Fig. 2:**
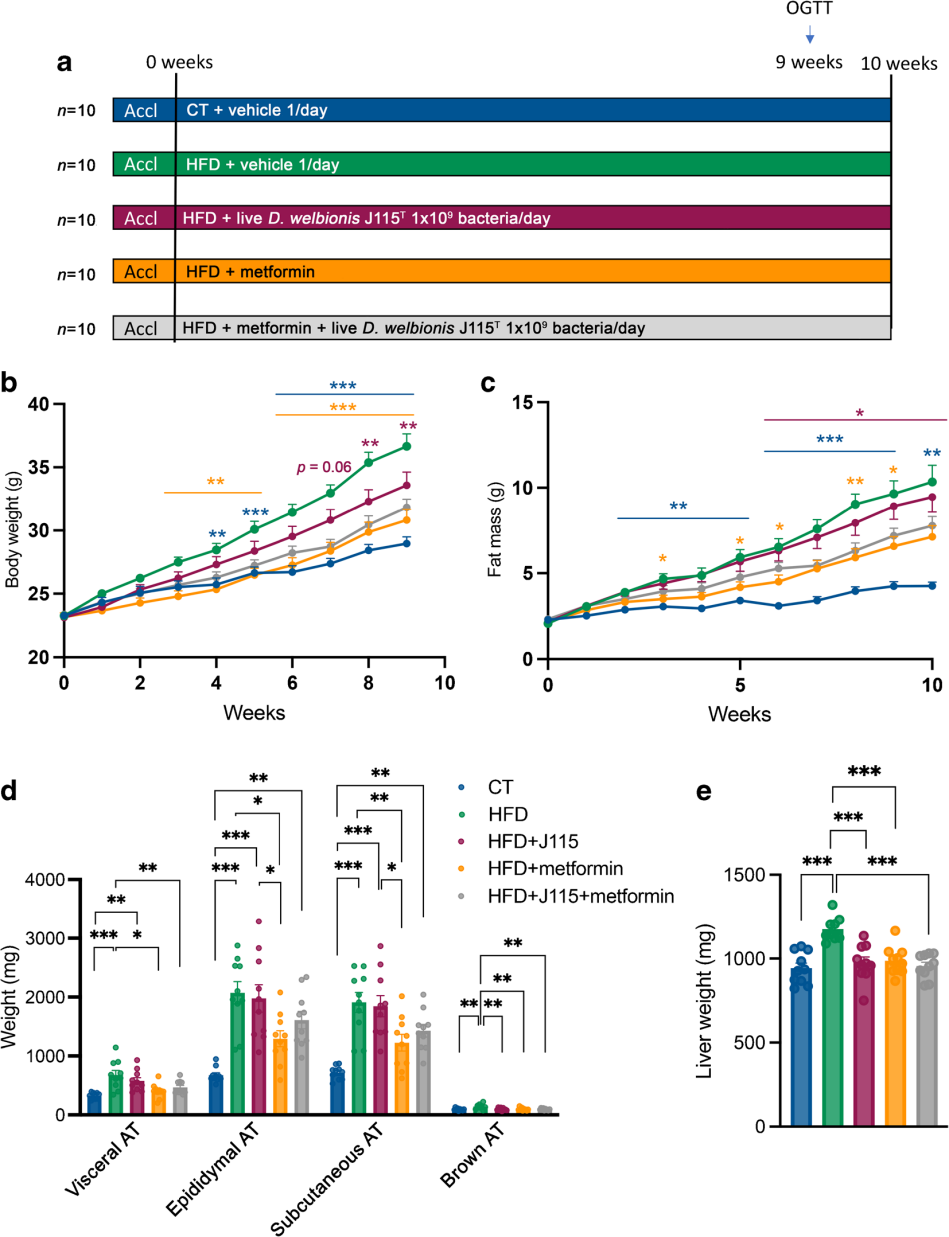
Metformin and *D. welbionis* J115^T^ treatment reduces diet-induced obesity, fat mass accumulation and liver weight without synergistic effects. (**a**) Experimental design of the study. (**b**, **c**) Body weight and fat mass evolution during 10 weeks of mice being fed a normal diet (CT) or HFD, treated with either a daily oral gavage of vehicle or live *D. welbionis* J115^T^ (HFD+J115) (1×10^9^ colony forming units [CFUs]), or of HFD mice treated with metformin (HFD+metformin) or a combination of metformin and live *D. welbionis* J115^T^ (HFD+J115+metformin). (**d**) Weight of the white adipose tissues (ATs) at the end of the 10 weeks: visceral AT, epididymal AT, subcutaneous AT and brown AT. (**e**) Liver weight. Number of mice per group: 9 (HFD) or 10 (other groups). Data were analysed using one-way ANOVA followed by Tukey’s post hoc test for parts (**d**) and (**e**) and two-way ANOVA followed by Tukey’s post hoc test for parts (**b**) and (**c**). *p<0.05; **p<0.01; ***p<0.001. Results are presented as bar plots with mean±SEM for parts (**d**) and (**e**). In parts (**b**) and (**c**), blue asterisks are for comparisons between the HFD and CT groups, orange asterisks are for comparisons between the HFD and HFD+metformin groups and maroon asterisks and the maroon *p* value are for comparisons between the HFD and HFD+J115 groups groups. Accl, acclimatisation

To further explore whether the effects of metformin and *D. welbionis* are intertwined, we designed an in vivo experiment in mice. We treated mice with HFD supplemented or not with metformin and with either *D. welbionis* J115^T^ or vehicle for 10 weeks (Fig. 2a). We confirmed that the administration of *D. welbionis* J115^T^ lowered HFD-induced body weight gain (Fig. 2b). Metformin was more efficient to limit HFD-induced body weight gain compared with *D. welbionis* J115^T^ (Fig. 2b), and the combination of metformin + *D. welbionis* J115^T^ had no additive effect. Except for a significantly lower BAT weight, the effects of *D. welbionis* J115^T^ on the reduction of fat mass gain were not statistically significant, whereas fat mass was significantly reduced in metformin-treated mice and visceral fat was significantly reduced when the combination was used (Fig. 2c,d). Untreated HFD-fed mice were characterised by a 20% higher liver weight compared with CT-fed mice (Fig. 2e). Treatment with *D. welbionis* J115^T^, metformin or the combination of both significantly lowered liver weight, which was normalised and equivalent to that of control lean mice (Fig. 2e). The lower liver weight was not explained by a change in total triacylglycerol or cholesterol content in livers of treated mice (ESM Fig. 2a,b). In addition, using qPCR we measured several markers of hepatic inflammation such as monocyte chemoattractant protein 1 (MCP1), LPS binding protein (LBP) and IL-1β (ESM Fig. 3a–c). HFD-treated mice did not display specific signs of hepatic inflammation.

### *D. welbionis* J115^T^ improves glucose tolerance to a similar extent to metformin

**Fig. 3:**
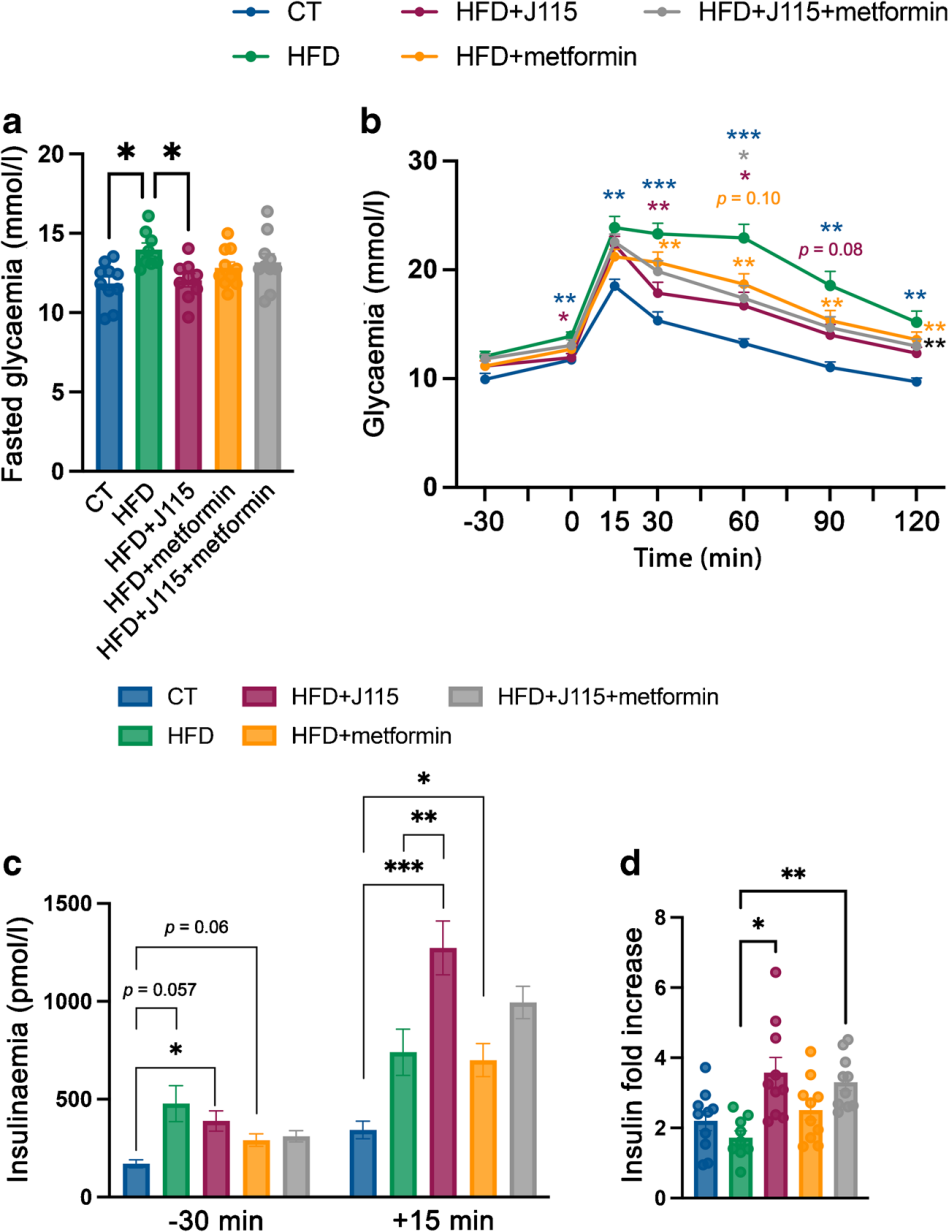
*D. welbionis* J115^T^ improves glucose tolerance to a similar extent to metformin. (**a**) Fasted glycaemia. (**b**) Plasma glucose profile during a 2 h OGTT. (**c**) Plasma insulin levels 30 min before and 15 min after glucose administration. (**d**) Insulin fold increase between +15 and −30. Number of mice per group: 9–10. Data were analysed using one-way ANOVA followed by Tukey’s post hoc test for parts (**a**), (**c**) and (**d**). *p<0.05; **p<0.01; ***p<0.001. Data were analysed using two-way ANOVA followed by Tukey’s post hoc test for part (**b**). *p<0.05; **p<0.01; ***p<0.001. Blue asterisks are for comparisons between the HFD and CT groups, maroon asterisks or maroon *p* value are for comparisons between the HFD and HFD+J115 groups, grey asterisks are for comparisons between the HFD and HFD+J115+metformin groups, orange *p* value is for comparison between the HFD and HFD+metformin groups, orange asterisks are for comparisons between the metformin and CT groups and black asterisks are for comparisons between HFD+J115 and CT groups. Results are presented as mean±SEM for parts (**a**), (**c**) and (**d**)

To further explore whether the lower body weight, liver mass and fat mass were associated with changes in glucose metabolism, we performed an OGTT. First, we found that *D. welbionis* J115^T^-treated mice exhibited significantly lower fasted blood glucose levels comparable to that of control lean mice, whereas this effect was not observed in metformin-treated groups (Fig. 3a). Mice receiving *D. welbionis* J115^T^ had significantly reduced glycaemia and a better OGTT profile than metformin-treated mice (Fig. 3b). To further explore the mechanism by which *D. welbionis* J115^T^ led to an improved glucose profile despite higher fat mass, we measured insulinaemia at baseline and 15 min after glucose administration. Fasting insulinaemia was higher in mice treated with HFD (280%), *D. welbionis* J115^T^ (220%) and metformin (160%) compared with CT mice (Fig. 3c). As expected, insulin levels were significantly increased 15 min after the oral glucose challenge in CT mice (>twofold increase) and to a lower extent in HFD mice (<twofold increase), although these results were not significant (Fig. 3d). This effect was significantly higher (about 3.5-fold increase) for mice receiving *D. welbionis* J115^T^ (HFD+J115 and HFD+J115+metformin). Consequently, *D. welbionis* J115^T^ treatment may partially resolve HFD-induced impaired glucose tolerance by stimulating glucose-induced insulin secretion. It is noteworthy that these effects were observed despite a higher insulin resistance index in all groups except metformin-treated mice (ESM Fig. 4).

### *D. welbionis* J115^T^ reduces fasting glycaemia by mechanisms independent of glucagon levels and gluconeogenesis markers

**Fig. 4:**
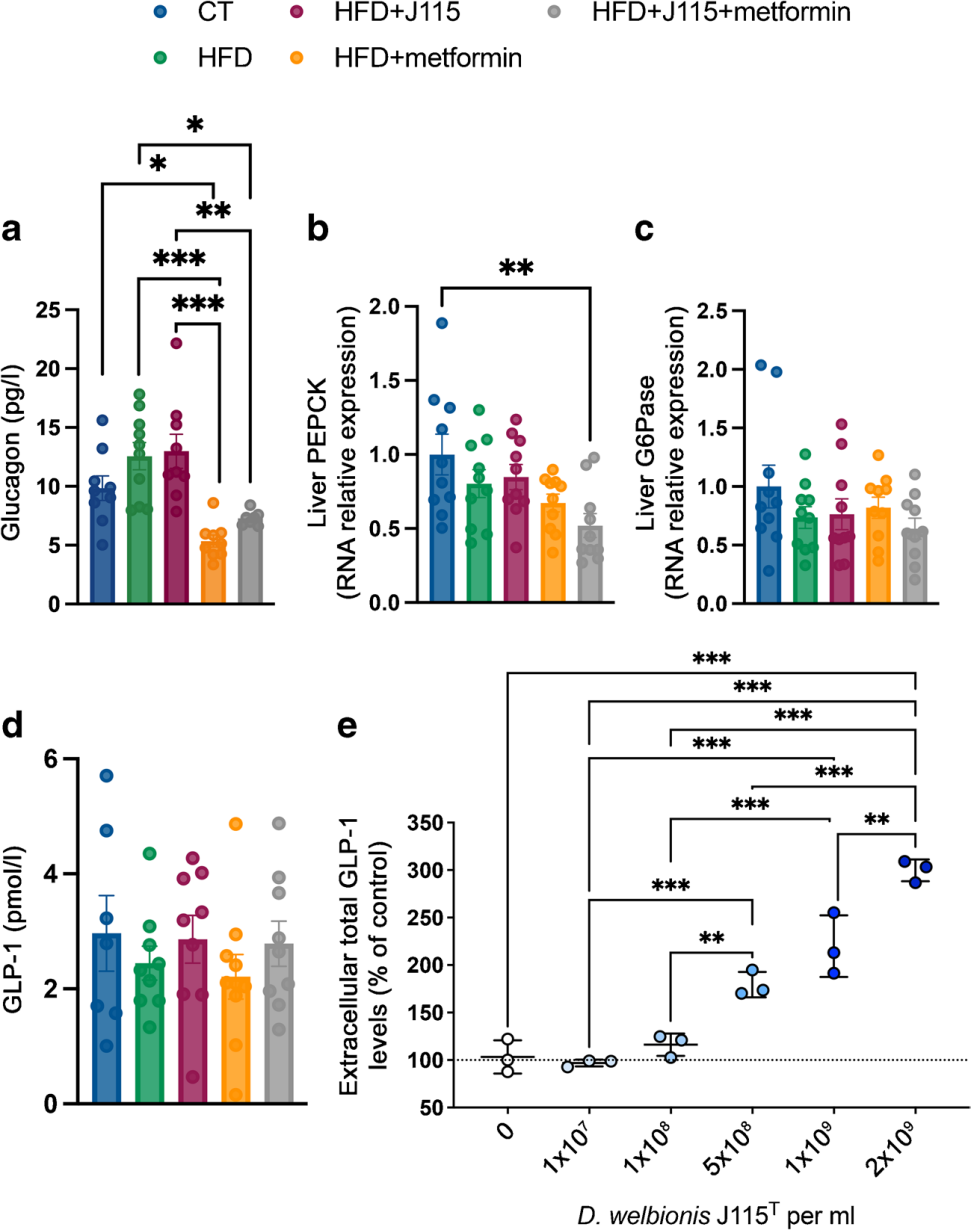
*D. welbionis* J115^T^ reduces fasted glycaemia by mechanisms independent of glucagon levels and hepatic gluconeogenesis markers. (**a**) Portal vein glucagon levels. (**b**, **c**) mRNA expression of *PEPCK* and *G6Pase* genes relative to the control group. (**d**) Portal vein GLP-1 levels. (**e**) Effect of different doses of live *D. welbionis* J115^T^ (HFD+J115) (from 1×10^7^ to 2×10^9^ colony forming units [CFUs] per ml) on GLP-1 stimulation in GLUTag cells (murine L cells). Data were analysed using one-way ANOVA followed by Tukey’s post hoc test for all figure parts. *p<0.05; **p<0.01; ***p<0.001. Results are presented as mean±SEM

To further decipher the potential mechanisms by which *D. welbionis* J115^T^ acts on fasting glycaemia, glucose tolerance and insulin levels, we measured hormones involved in glucose metabolism. The lower fasting glycaemia in *D. welbionis* J115^T^-treated mice was explained neither by fasting glucagon levels (Fig. 4a) nor by the mRNA expression of key liver gluconeogenic enzymes, that is, PEPCK and glucose 6-phosphatase (G6Pase) (Fig. 4b,c, ESM Table 1). Metformin significantly lowered fasting glucagon levels in HFD mice (Fig. 4a), independently of *PEPCK* and *G6Pase* mRNA expression (Fig. 4b,c). We asked whether the higher insulin secretion upon oral glucose load is related to the incretin GLP-1. We found that portal plasma levels of GLP-1 were not significantly different between groups (Fig. 4d). However, these levels were measured in portal vein blood of fasted mice at the end of the experiment; we did not have access to the portal vein during the OGTT.

To circumvent this, we used enteroendocrine L cells in vitro to assess whether *D. welbionis* J115^T^ directly promotes GLP-1 secretion (Fig. 4e). *D. welbionis* J115^T^ significantly stimulates secretion of GLP-1 in a dose-dependent manner, suggesting that the bacterium can communicate with enteroendocrine cells. It is worth noting that the levels of the active form of GLP-1 were also positively correlated with the levels of *D. welbionis* in the human cohort (ESM Fig. 5).

### *D. welbionis* J115^T^ and metformin reduce cumulative energy intake

**Fig. 5:**
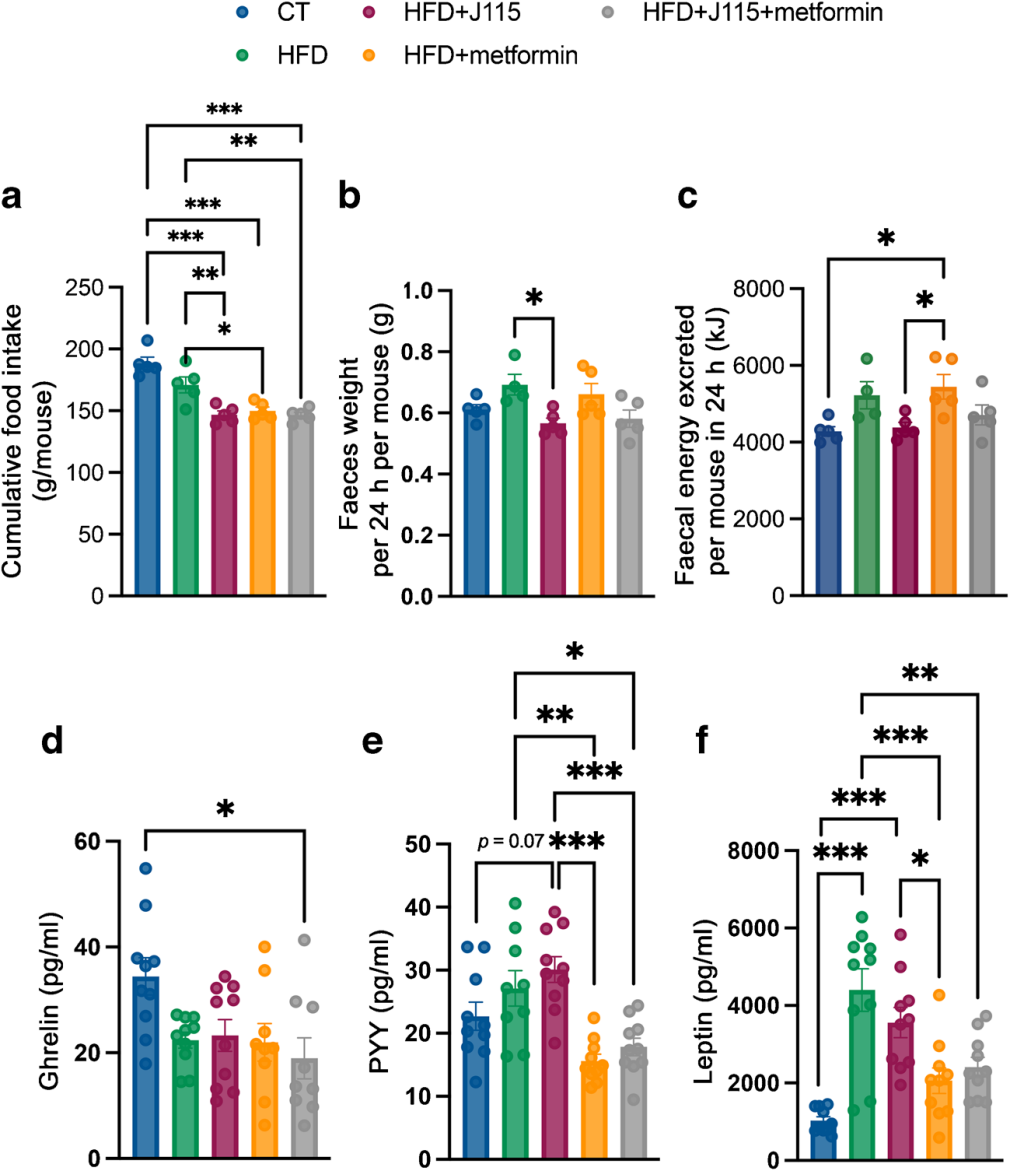
*D. welbionis* J115^T^ and metformin treatment reduces cumulative energy intake and changes gut peptides involved in appetite regulation. (**a**) Cumulative food intake. (**b**) Total faeces weight per 24 h per cage of two mice. (**c**) Faecal energy excreted per 24 h measured using bomb calorimetry. (**d**) Portal vein ghrelin levels. (**e**) Portal vein PYY levels. (**f**) Portal vein leptin levels. Number of mice per group: 9–10. Data were analysed using one-way ANOVA followed by Tukey’s post hoc test. *p<0.05; **p<0.01; ***p<0.001. Results are presented as mean±SEM

Next, we assessed whether the observed decreases in body weight and fat mass were associated with changes in energy intake. We found that both *D. welbionis* J115^T^- and metformin-treated mice consumed less food throughout the experimental period; the combination of both did not amplify this effect (Fig. 5a). Because metformin-treated mice gained less weight and fat mass than *D. welbionis* J115^T^-treated mice despite similar energy intake, we investigated whether part of the energy consumed was excreted via the faeces. We found that *D. welbionis* J115^T^-treated mice excreted less faeces per 24 h than HFD mice (Fig. 5b), while metformin did not affect this. Using bomb calorimetry, we determined that total energy excreted in the faeces was lower in *D. welbionis* J115^T^-treated mice compared with HFD-fed metformin-treated animals (Fig. 5c). To explain the lower food intake observed in both *D. welbionis* J115^T^- and metformin-treated mice, we measured hormones involved in the regulation of appetite such as the orexigenic hormone ghrelin and anorexigenic PYY. We found that ghrelin was decreased similarly in all mice on HFD (Fig. 5d), but that PYY levels were highest in *D. welbionis* J115^T^-treated mice not receiving metformin (Fig. 5e). Leptin is considered an anorexigenic hormone but mostly reflects the adiposity of the mice. Leptin levels were significantly increased in HFD mice compared with CT. Metformin reduced leptin levels, while *D. welbionis* J115^T^ did not (Fig. 5f).

### *D. welbionis* J115^T^ abolishes HFD-induced adipocyte hypertrophy and whitening of BAT

**Fig. 6:**
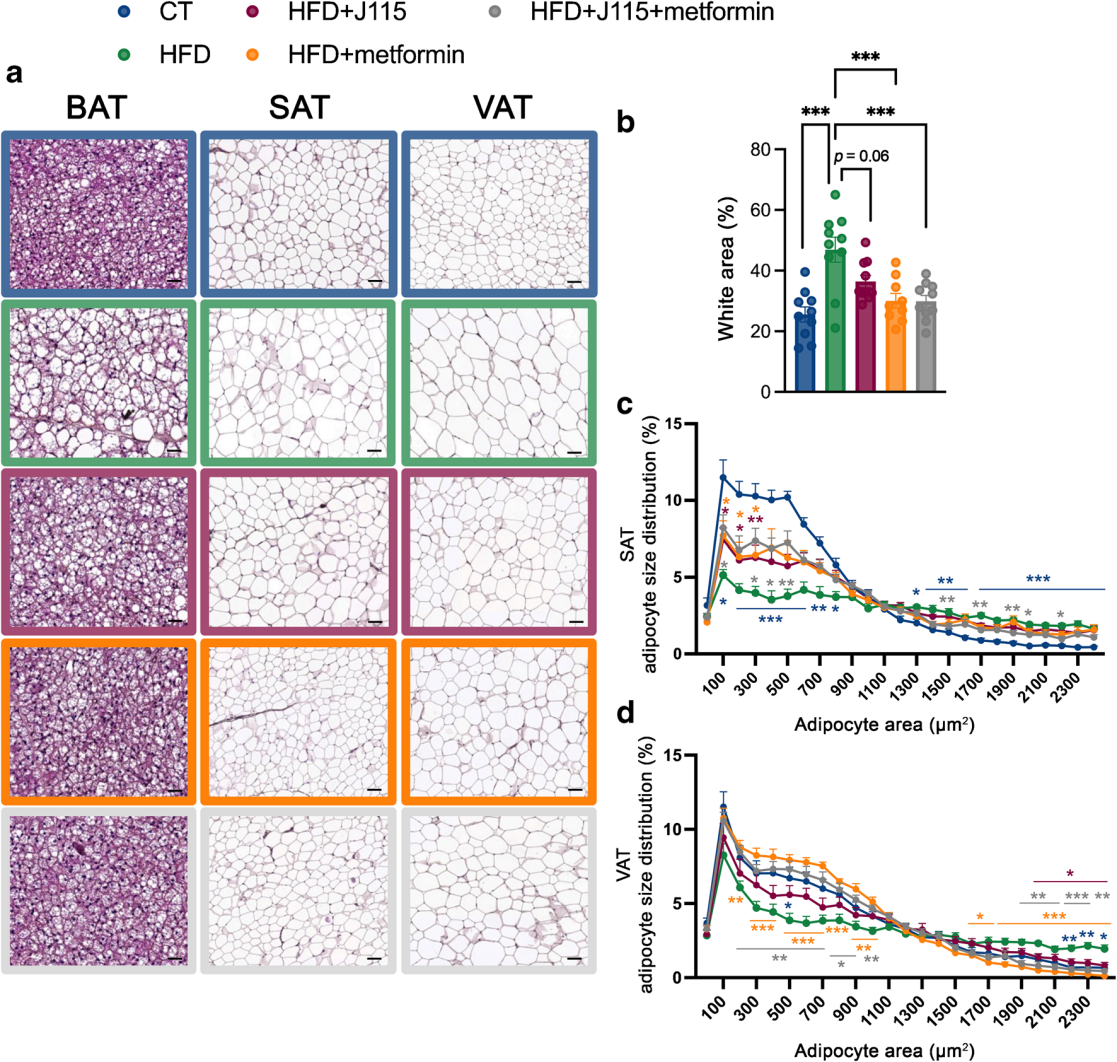
*D. welbionis* J115^T^ abolishes HFD-induced adipocyte hypertrophy and the whitening of BAT. (**a**) Representative H&E-stained pictures of BAT and subcutaneous and visceral adipose tissues (SAT and VAT, respectively). (**b**) Percentage of white area on the slices, corresponding to lipid droplets, in the BAT. (**c**) Adipocyte diameter (μm) distribution in the SAT. (**d**) Adipocyte diameter (μm) distribution in the VAT. Number of mice analysed per group: 9–10. Number of pictures per animal per tissue: 5. Data were analysed using one-way ANOVA followed by Tukey’s post hoc test. *p<0.05; **p<0.01; ***p<0.001. Blue asterisks are for comparisons between the HFD and CT groups, grey asterisks are for comparisons between HFD and HFD+metformin+J115 groups, orange asterisks are for comparisons between the metformin and CT groups and maroon asterisks are for comparisons between HFD+J115 and CT groups. Results are presented as mean±SEM. SAT and VAT: scale bar, 50 µm; BAT: scale bar, 25 µm

We found previously that *D. welbionis* J115^T^ decreases whitening of BAT, thereby contributing to a better functionality of BAT and to oxidation of nutrients [19]. Here, *D. welbionis* J115^T^ also reduced whitening of BAT, but this did not reach significance (*p*=0.06) (Fig. 6a,b). *D. welbionis* J115^T^ does not decrease fat mass to the same extent as metformin (Fig. 2c,d). Despite this limited impact on total fat mass, it had major effects on fasting glycaemia, OGTT glycaemic profile and insulin secretion. Given that smaller adipocytes are known to be more insulin sensitive and to contribute to lower glucose levels compared with hypertrophic adipocytes [22, 23], we measured mean adipocyte size as well as the adipocyte size distribution in subcutaneous and visceral (white) adipose tissues. We found that HFD mice had more large adipocytes and fewer small adipocytes in both white fat depots when compared with CT mice (Fig. 6a,c,d), suggesting that HFD-induced fat cell hypertrophy. Conversely, despite harbouring almost the same total fat mass as HFD mice (Fig. 2b), *D. welbionis* J115^T^-treated mice had a significantly different size distribution profile compared with HFD-fed mice, with lower frequency of large adipocytes and higher occurrence of small adipocytes (Fig. 6a,c,d) in both subcutaneous and visceral fat depots. Similar profiles were observed in mice treated with metformin.

## Discussion

In this study, we present novel findings regarding the abundance of *D. welbionis* and its relationship with prebiotic treatment and metformin administration. In a cohort involving 106 participants with obesity, including both men and women, in which we investigated the abundance of *D. welbionis* in faeces before and after prebiotic treatment and in the presence or absence of metformin, we found that *D. welbionis* was unaffected by prebiotic treatment, but was significantly more abundant in metformin-treated participants. Additionally, *D. welbionis* was negatively correlated with hepatic toxicity markers in humans with diabetes and obesity, and with fasting glycaemia in individuals treated with metformin. Our study further revealed that the abundance of *D. welbionis* was linked to response to prebiotic treatment. Responders had nearly three times higher levels of *D. welbionis* in their faeces before starting the treatment, suggesting a correlation between microbiota composition and efficacy of prebiotic treatment.

In vitro experiments showed that the growth of *D. welbionis* J115^T^ was not directly affected by metformin, suggesting that the increase in *D. welbionis* observed in the human gut is likely due to a complex microbial interaction, such as crossfeeding. To demonstrate causality, we performed in vivo experiments, showing that *D. welbionis* J115^T^ reduced diet-induced obesity and liver weight. The combination of metformin and *D. welbionis* J115^T^ did not show additive or synergistic effects. Nevertheless, in accordance with previous studies [19], mice treated with *D. welbionis* J115^T^ exhibited an improved glucose profile which we linked to increased glucose-induced insulin secretion. The lower fasting glycaemia was explained neither by fasting glucagon levels nor by key gluconeogenic enzymes in the liver. *D. welbionis* J115^T^ significantly and dose-dependently stimulated GLP-1 secretion in vitro, suggesting that the bacterium communicates with enteroendocrine cells and could thereby affect food intake. Indeed, we found that *D. welbionis* J115^T^-treated mice consumed less food. Interestingly, mice receiving metformin alone consumed equal amounts of food as *D. welbionis* J115^T^-treated mice but excreted more energy via the faeces, probably explaining some of the differences in fat mass gain. However, this is not sufficient to explain all the differences in fat mass gain, as mice receiving both treatments did not excrete more energy in kJ.

Similarly, we found in humans that *D. welbionis* abundance was positively correlated with PYY and GLP-1 levels (ESM Fig. 5). We previously discovered that *D. welbionis* J115^T^ produces SCFAs such as butyrate and propionate. These SCFAs have been shown to stimulate the secretion of gut peptides such as PYY and GLP-1 by activating G protein-coupled receptors such as G protein-coupled receptor 41 (GPR41) and GPR43. Whether the higher GLP-1 and PYY in humans is due to a possible SCFAs*–D. welbionis* J115^T^-dependent mechanism remains speculative. Of note, in enteroendocrine cells we found a direct dose-dependent stimulation of GLP-1 secretion, suggesting that the effects could be due to bacterial compounds rather than the presence of SCFAs in the culture medium. Further investigations are needed to decipher the exact molecule or group of molecules produced by *D. welbionis* J115^T^, as previously done for other beneficial bacteria [6, 24–26].

In this study, the lower body weight gain observed in *D. welbionis* J115^T^-treated mice was not fully reflected by a lower fat mass. One possible explanation would be a difference in fat-free mass. However, we have not observed any difference between metformin- and *D. welbionis* J115^T^-treated mice for this variable (not shown). Another possible explanation could be a lower glycogen content in the bodies of *D. welbionis* J115^T^-treated mice. This hypothesis warrants further investigation.

Despite the differences in fat mass, both metformin and *D. welbionis* J115^T^ significantly decreased the whitening of BAT. We previously discovered that *D. welbionis* J115^T^ also increases the number and activity of mitochondria in this tissue, an effect associated with lower inflammation [19]. Although metformin and *D. welbionis* J115^T^ similarly reduced the formation of white-like adipocytes in BAT, the impact on total white fat mass was discrepant between treatments. We cannot rule out that the lowering of white fat mass does not fully rely on BAT activity. For instance, metformin has been shown to modulate gut microbiota in favour of specific bacteria such as *Subdoligranulum* and *A. muciniphila* [5, 13, 27, 28]. Whether treatment with *D. welbionis* J115^T^ or metformin differentially affects the gut microbiota was not explored in this study.

Besides metformin, smoking and physical activity might be additional confounding factors. In this study, no relationship between the levels of *Dysosmobacter* and smoking status were observed. Interestingly, in this group of individuals, a specific set of participants (*n*=61) were recruited from two of the three hospitals [29]. They completed an International Physical Activity Questionnaire (IPAQ) at both the beginning and the conclusion of the protocol to assess their physical activity levels throughout the study. The participants were not given any guidance to alter their physical activity habits during the research period. At the conclusion of the study, no relationship with *Dysosmobacter* was observed for low-, moderate- or high-intensity or overall physical activity level for the participants in either cohort (with 31 individuals in the maltodextrin group and 30 individuals in the inulin group).

Overall, this study contributes to our understanding of the relationship between gut microbiota composition and prebiotic and metformin treatments. The strengths of this study rely on the translational aspects of our work. We obtained data using in vitro and in vivo approaches in mice, humans and cell lines. Moreover, the current data highlight that specificities of the gut microbiota drive the metabolic and microbial responses to inulin.

Among the limitations, we were unable to fully dissect the direct role of metformin on *Dysosmobacter* levels; this will require an appropriate design using samples obtained before and after supplementation with metformin. As discussed, other possible confounding factors exist in human studies such as smoking, the use of specific drugs (e.g. metformin, proton pump inhibitors, antibiotics), physical activity, food intake and others. In this study, some of these variables were controlled either during the selection or after statistical correction. However, whether *Dysosmobacter* could be influenced directly by smoking status, specific drugs or dietary items other than inulin is unknown at this stage. Finally, the mouse study was performed in male mice because they are more prone to developing metabolic disorders than female mice. However, further studies should be done to delineate possible sex differences.

In conclusion, our data show that *D. welbionis* J115^T^ was more potent than metformin, the gold standard comparator in the field, for glucose tolerance and had beneficial effects on insulin secretion. These findings may have implications for the development of personalised approaches for the treatment of obesity and diabetes.

### Supplementary Information

Below is the link to the electronic supplementary material.

Supplementary file1 (PDF 591 KB)

## Data Availability

All the data contained within this article are available within the paper. All the participant characteristics and markers measured in the human trial are available in Rodriguez et al [14] and Hiel et al [16]. The trial protocol was published on protocols.io (dx.doi.org/10.17504/protocols.io.baidica6). The raw data on mice will be shared upon reasonable request: Contact Patrice D. Cani (UCLouvain, patrice.cani@uclouvain.be).

## Abbreviations

ALT: Alanine aminotransferase
AST: Aspartate aminotransferase
BAT: Brown adipose tissue
CT: Control diet
GLP-1: Glucagon-like peptide-1
G6Pase: Glucose 6-phosphatase
HFD: High-fat diet
MSD: Meso Scale Discovery
PYY: Peptide YY
qPCR: Quantitative PCR
SCFA: Short-chain fatty acid
