# Supplementary material for: Impact of metformin and Dysosmobacter welbionis on diet-induced obesity and diabetes: from clinical observation to preclinical intervention
Source: Diabetologia. 2023 Oct 28;67(2):333–45. doi: 10.1007/s00125-023-06032-0 (PMC10789671; doi:10.1007/s00125-023-06032-0)
Supplement: Supplementary file 1 — Supplementary file1 (PDF 591 KB) [file 125_2023_6032_MOESM1_ESM.pdf]

ELECTRONIC SUPPLEMENTARY MATERIAL (ESM)

ESM Methods

Human cohort

Male and female subjects (106 subjects) were recruited in three university hospitals in Belgium (Cliniques universitaires Saint-Luc and Hôpital Erasme in Brussels, and Centre Hospitalier Universitaire in Liège) as previously described [1-3]. All participants were blinded during the entire trial. The inclusion criteria were a BMI > 30 kg/m2, subjects aged from 18 to 65 years, Caucasian ethnicity and the presence of at least one obesity-related metabolic disorder (i.e., prediabetes or type 2 diabetes, dyslipidemia, hypertension, elevated γ-glutamyl transferase (gGT) and/or alanine aminotransferase (ALT) and/or aspartate aminotransferase (AST)). Subjects were randomly assigned to either the prebiotic or the placebo arm and the randomization sequences were not revealed to the study staff. The exclusion criteria included the use of probiotics, prebiotics, antibiotics, dietary fibers supplements, or any molecules that modifies the gut microbiota composition such as following a special diet (e.g., vegetarian, vegan), any recent (<6 weeks) or ongoing diets (e.g., rich in protein, rich in fiber), alcohol consumption higher than 3 glasses/day), type 1 diabetes, the recent use (<6 weeks) of supplements or drugs affecting the transit time within, pregnancy in progress or planned within 6 months, the presence of a psychiatric problem and/or the use of antipsychotic drugs. This study was approved by the “Comité d’éthique Hospitalo-facultaire de Saint-Luc”. Written informed consent was obtained from all participants before inclusion. The trial protocol, named Food4gut, was published on protocols.io (dx.doi.org/10.17504/protocols.io.baidica6) and the trial was registered at ClinicalTrials.gov under identification number NCT03852069.

Participants were included for a period of three months and randomized to consume either 16g/d of native inulin (extracted from chicory root, Cosucra, Belgium) or 16 g/d of maltodextrin (Cargill, Belgium), provided in identical packaging. Empty and unused packets were returned to measure compliance. To promote adaptation to the fiber, patients were asked to ingest half the dose during the first week. Participants received a cookbook with recipes based on vegetables either rich or poor in fructans and were advised to consume at least one meal proposed in the recipe book per day.

Stools samples were collected at baseline and at the end of the 3-month of intervention and stored at room temperature with a DNA stabilizer (Stratec biomolecular, Berlin, Germany) for maximum three days, then transferred to -80°C. Genomic DNA was extracted from faeces using a PSP® spin stool DNA kit (Stratec biomolecular, Berlin, Germany).
Blood samples were collected using BD^{TM} P800 Collection and Preservation System, which contains DPP-IV and other protease inhibitors. Plasma concentrations of Fibroblast growth factor 21 (FGF21), active ghrelin, total glucagon-like peptide 1 (GLP-1), glucagon, leptin, pancreatic polypeptide (PP), total peptide YY (PYY) were determined using the Meso Scale Discovery (MSD) U-PLEX assay (Rockville, MD, USA) following the manufacturer’s instructions. Analyses were performed using a QuickPlex SQ 120 instrument (MSD) and DISCOVERY WORKBENCH® 4.0 software (MSD, Rockville, MD, USA).

qPCR quantification of Dysosmobacter welbionis and total bacteria in human samples

Absolute quantification of the total bacterial load was performed by quantitative polymerase chain reaction (qPCR) using the primers Bacteria Universal P338F (ACTCCTACGGGAGGCAGCAG) and P518R (ATTACCGCGGCTGCTGG) as previously described in [4]. Real-time PCR was performed with a QuantStudio3 (Applied Biosystems, The Netherlands) using SYBR Green (GoTaq® qPCR mix, Promega, USA) for detection and using the QuantStudio Software (Version 1.4.3, Applied Biosystems, The Netherlands). Absolute quantification was achieved through the inclusion of a standard curve (performed in duplicate) on each plate generated by diluting DNA from pure culture of L. acidophilus NCFM (five-fold serial dilution). Cell counts were determined by plating and expressed as “colony-forming unit” (CFU) before DNA isolation.

Animal ethics

All mouse experiments were approved by the Ethical Committee for Animal Care of the Health Sector of the Université Catholique de Louvain (UCLouvain) headed by Prof. J-P Dehoux, under number 2022/UCL/MD/09, and were performed in accordance with the guidelines of the Local Ethics Committee and in accordance with the Belgian Law of 29 May 2013, regarding the protection of laboratory animals (agreement number LA1230314)

Culture and preparation of *Dysosmobacter welbionis* for mouse experiments

D. welbionis J115^T was cultured anaerobically in a modified YCFA medium supplemented with 10 g/L inositol. Cultures were centrifuged at 5000g during 15 min and the supernatant was removed. Cells were then resuspended in anaerobic PBS-carbonate buffer supplemented with 15 % (vol/vol) trehalose, then immediately frozen in anaerobic vials and stored at -80°C. The number of total and cultivable bacteria administered to the mice was calculated by plating the bacterial culture before preparation and the bacterial suspension after preparation for administration.

Mouse model

Sets of 7-week-old C57BL/6J male mice (Janvier Laboratories, Le Genest-Saint-Isle, France) were housed in pairs in SOPF (specific opportunistic and pathogen free) conditions, in a controlled environment (room temperature of 22 ± 2°C, 12h daylight cycle) with free access to sterile (irradiated) food and sterile (autoclaved) water. Upon arrival, mice were randomly separated at 2 animal/cage and underwent a 1-week acclimatization period, during which they were fed a control diet (CT (AIN93Mi, Research Diet, New Brunswick, NJ, USA). During the experiments, food and water intake were recorded once a week. Body composition was assessed by using a 7.5 MHz time domain-nuclear magnetic resonance machine (TD-NMR) (LF50 minispec, Bruker, Rheinstetten, Germany).

A set of 50 mice was divided in 5 groups of 10 mice. The mice were fed either control diet (CT, AIN93Mi) or high-fat diet (HFD, 60% fat and 20% carbohydrates (kcal/100g), D12492, Research diet). The HFD+J115 group was given *Dysosmobacter welbionis* J115^T by oral gavage at the dose 1.10^9 cfu/0.2 ml per day and per mice. The HFD+metformin group was orally administered 150 mg/kg/day the first 2 weeks and 100 mg/kg/day for the rest of the study. In the group HFD+J115+metformin, mice were treated with joint administration of *Dysosmobacter welbionis* J115^T and metformin by daily oral gavage. In the control groups, control diet (CT) and HFD mice were treated with an oral gavage of an equivalent volume of PBS-carbonate buffer supplemented with 15% (weight/vol) trehalose. The treatment continued for 10 weeks. Mice were killed after a 6-hour fasting period.

Oral glucose tolerance test (OGTT)

One week before the end of the experiment, mice were fasted for 6 hours before being given an oral gavage glucose load (2 g glucose per kg body weight). Blood glucose was measured 30 minutes before (timepoint -30), just prior to the oral glucose load (timepoint 0) and then after 15, 30, 60, 90 and 120 min. Blood glucose was determined with a glucometer (Accu Chek Performa, Roche, Basel, Switzerland) on blood samples collected from the tip of the tail vein. Plasma insulin concentration was determined by ELISA (Mercodia, Uppsala, Sweden) according to the manufacturer's instructions.

Tissue sampling

The animals were anesthetized with isoflurane (Forene®, Abbott, Queenborough, Kent, England) and blood was collected from the portal and cava veins. Then mice were killed by decapitation. Tissue samples (liver, brown adipose tissue, subcutaneous adipose tissue, mesenteric adipose tissue) were dissected, immersed in liquid nitrogen, and stored at –80 °C for further analysis. A part of the adipose tissues was fixed in 4 % paraformaldehyde in PBS for histological analysis.

Insulin resistance index

The insulin resistance index was determined by multiplying the area under the curve (from −30 to 15 min) of blood glucose and plasma insulin obtained during the OGTT.

Histological analyses

Brown and white adipose tissues were fixed in 4 % paraformaldehyde for 24 h at room temperature. Samples were then dehydrated by immersion in ethanol 100% for 24 h and processed for paraffin embedding. Paraffin sections of 5 µm were stained with haematoxylin and eosin. Whole tissue sections were digitalized using a Panoramic ScanII slide scanner (3DHistech Ltd, Budapest, Hungary) with a 20x Plan-Apochromat objective and visualized with the Cytomine web platform. The white area in brown adipose tissue corresponding to (empty) lipid droplets were quantified based on 5 fields per sample using Fiji software. The adipocyte size and frequency in SAT and VAT was assessed using Adiposoft [5] on Fiji software. 5 pictures per adipose tissue were taken. Mean number of adipocytes per picture: CT: 400, HFD: 200, HFD+J115/HFD+metformin/HFD+J115+metformin: 300 depending on the adipocyte size at this magnification.

Gene expression analysis by real-time qPCR

Total RNA was prepared from tissues using TriPure reagent (Roche). Quantification and integrity analysis of total RNA was performed by running 1 µl of each sample on an Agilent 2100 Bioanalyzer (Agilent RNA 6000 Nano Kit, Agilent).

For qPCR analysis, cDNA was prepared by reverse transcription of 1 µg total RNA using a Reverse Transcription System kit (Promega, Leiden, The Netherlands). Real-time PCRs were performed with the StepOnePlus real-time PCR system and software (Applied Biosystems, Den Ijssel, The Netherlands) using GoTaq® qPCR mix, (Promega, USA) for detection, according to the manufacturer's instructions. RPL19 was chosen as the reference gene. All samples were run in duplicate in a single 96-well reaction plate, and data were analyzed according to the $2^{-\Delta\Delta Ct}$ method. The identity and purity of the amplified product were checked through analysis of the melting curve at the end of amplification. Primer sequences for the mouse genes are shown in supplemental ESM Table 1.

Plasma Multiplex Analysis in mouse plasma

Plasma levels of total GLP-1, total PYY, total ghrelin, leptin and glucagon were measured from the portal vein by multiplex assay kits based on chemiluminescence detection, following manufacturer's instructions (Meso Scale Discovery (MSD), Rockville, MD, USA). Analyses were performed using a QuickPlex SQ 120 instrument (MSD) and DISCOVERY WORKBENCH® 4.0 software (MSD, Rockville, MD, USA).

Statistical analysis

Statistical analyses were performed using GraphPad Prism version 9.4.0 (GraphPad Software, San Diego, CA, USA) and RStudio 2022.07.1 for MacOS. For figure 1a: Two-way ANOVA was performed followed by Sidak multiple comparisons at M0 and M3. For figure 1b, Two-tailed unpaired T-test was performed. For figure 1c and figure 2 to 6: Comparison between three or more groups on one time-point was performed by one-way ANOVA followed by Tukey's multiple comparison test. Comparison between 3 or more groups at different time-points was performed by two-way repeated measures ANOVA followed by Tukey's multiple comparison test. $p < 0.05$ was considered statistically significant. The presence of outliers was assessed using ROUT's outlier test on GraphPad Prism. Figure 1d-e and S2: We used Rstudio program to perform the correlation matrices using *tidyverse*, *dplyr*, *ggplot2*, *corrr*,

corrplot, *Hmisc* and *psych* packages. Correlation analyses were assessed by Spearman's correlation tests, followed by a Holm's adjustment for multiple testing. Data are presented as means±SEM

GLP-1 stimulation on murine L cells (GLUTag cells)

GLUTag cells were kindly donated by Prof. Daniel Drucker (University of Toronto). The enteroendocrine L cell line maintenance and culture (without mycoplasma) was performed as previously described [6, 7]. For GLP-1 secretion studies, cells were plated on 1% Matrigel-coated 24-well plates and were allowed to adhere for 24 h. *D. welbionis* J115T cells were suspended in the cell line medium at increasing concentrations (from $1x10^7$ to $2x10^9$/ml) and incubated at 37 °C for 2 h in presence of DPP-IV inhibitor at 50 µM final concentration. Total GLP-1 levels in collected supernatants and cell lysates were measured using Meso Scale Discovery (MSD) ELISA kit. The GLP-1 release were calculated from the GLP-1 concentration measured in supernatant divided by total levels in the supernatant and the lysate.

References

[1] Rodriguez J, Hiel S, Neyrinck AM, et al. (2020) Discovery of the gut microbial signature driving the efficacy of prebiotic intervention in obese patients. Gut

[2] Hiel S, Gianfrancesco MA, Rodriguez J, et al. (2020) Link between gut microbiota and health outcomes in inulin -treated obese patients: Lessons from the Food4Gut multicenter randomized placebo-controlled trial. Clinical nutrition (Edinburgh, Scotland) 39: 3618-3628

[3] Neyrinck AM, Rodriguez J, Zhang Z, et al. (2021) Prebiotic dietary fibre intervention improves fecal markers related to inflammation in obese patients: results from the Food4Gut randomized placebo-controlled trial. Eur J Nutr

[4] Le Roy T, Moens de Hase E, Van Hul M, et al. (2022) Dysosmobacter welbionis is a newly isolated human commensal bacterium preventing diet-induced obesity and metabolic disorders in mice. Gut 71: 534-543

[5] Galarraga M, Campion J, Munoz-Barrutia A, et al. (2012) Adiposoft: automated software for the analysis of white adipose tissue cellularity in histological sections. J Lipid Res 53: 2791-2796

[6] Xu Y, Carradori D, Alhouayek M, et al. (2018) Size Effect on Lipid Nanocapsule-Mediated GLP-1 Secretion from Enteroendocrine L Cells. Mol Pharm 15: 108-115

[7] Xu Y, Van Hul M, Suriano F, Preat V, Cani PD, Beloqui A (2020) Novel strategy for oral peptide delivery in incretin-based diabetes treatment. Gut 69: 911-919

ESM Table 1. Primers for qPCR

Rpl-19	Forward<br>Reverse	GAAGGTCAAAGGGAATGTGTTCA<br>CCTGTTGCTCACTTGT
G6Pase<br>(glucose-6-phosphatase)	Forward<br>Reverse	AGGAAGGATGGAGGAAGGAA<br>TGGAACCAGATGGGAAAGAG
IL1b	Forward<br>Reverse	TCGCTCAGGGTCACAAGAAA<br>CATCAGAGGCAAGGAGGAAAAC
MCP1	Forward<br>Reverse	GCAGTTAACGCCCCACTCA<br>TCCAGCCTACTCATTGGGATCA
LBP	Forward<br>Reverse	GTCCTGGGAATCTGTCCTTG<br>CCGGTAACCTTGCTGTTGTT
PEPCK<br>(phosphoenolpyruvate carboxykinase 1)	Forward<br>Reverse	ACCTCCTGGAAGAACAAGGA<br>CTCATGGCTGCTCCTACAAA
Dysosmobacter welbionis J115^T	Forward<br>Reverse	ATACCGCATGACGCATGACC<br>CCAGCGATAAAATCTTTGACATGCC

ESM Figures

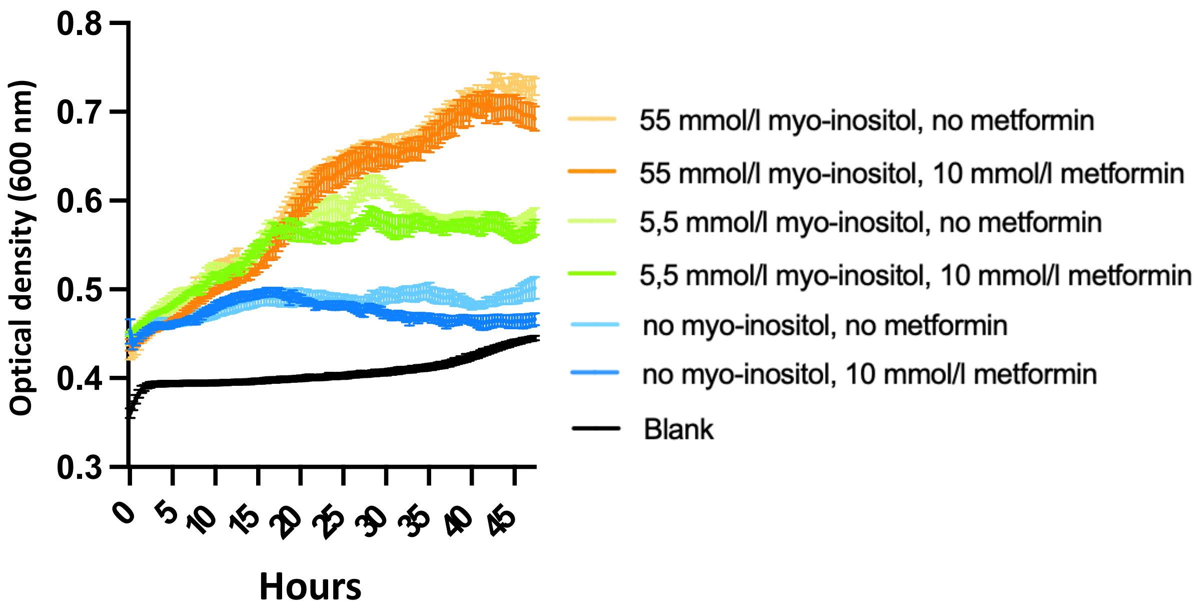

ESM Fig. 1. Growth curves of *D. welbionis* J115^{T} measured by optical density (OD) at 600 nm

Culture medium containing either 0, 5,5 or 55 mmol/L of myo-inositol and 0 or 10 mmol/L of metformin. n=5 per condition.

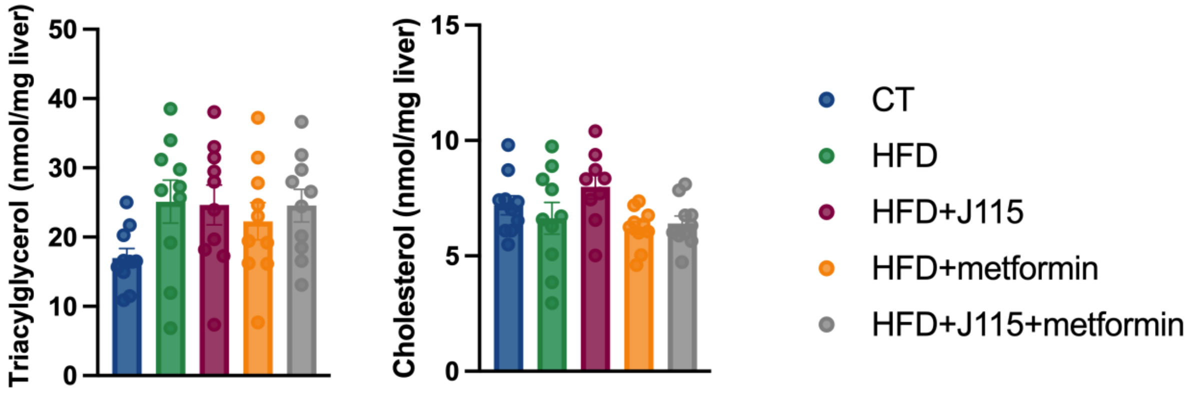

ESM Fig. 2. Triacylglycerol and cholesterol content in the liver of treated mice

(a) Triacylglycerol content (nmol/mg of liver), (b) Cholesterol content (nmol/mg of liver) in the treated mice. Number of mice analyzed per group: 10. Data were analyzed using one-way analysis of variance (ANOVA) followed by Tukey's post hoc test. Results are represented as mean±SEM.

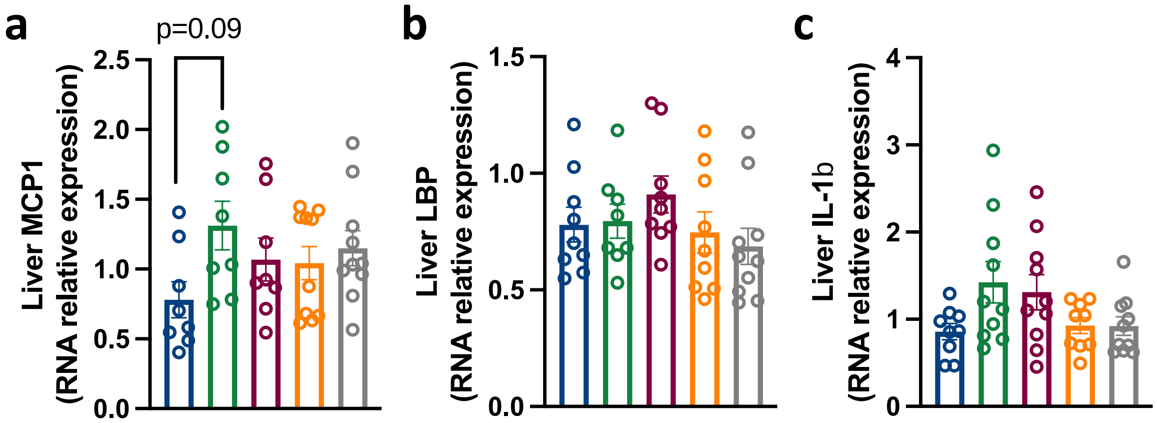

ESM Fig. 3. mRNA expression of inflammatory markers in the liver of treated mice

(a) mRNA expression of Monocyte Chemoattractant Protein 1 (MCP1), (b) LPS binding protein (LBP), (c) interleukin 1β (IL-1β) genes relative to the control group. Number of mice analyzed per group: 8-10. Data were analyzed using one-way analysis of variance (ANOVA) followed by Tukey's post hoc test. Results are represented as mean±SEM.

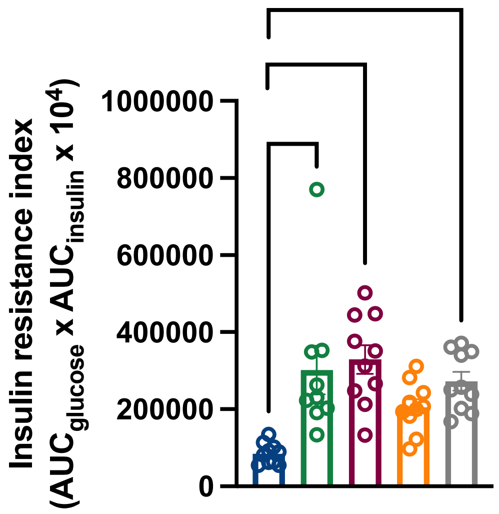

ESM Fig. 4. Insulin resistance index measured at the oral glucose tolerance test (OGTT)

Insulin resistance index determined by multiplying the area under the curve (from −30 to 15 min) of blood glucose and plasma insulin obtained during the OGTT. Number of mice per group: 9-10. Data were analyzed using one-way analysis of variance (ANOVA) followed by Tukey's post hoc test. **p<0.01; ***p<0.001.

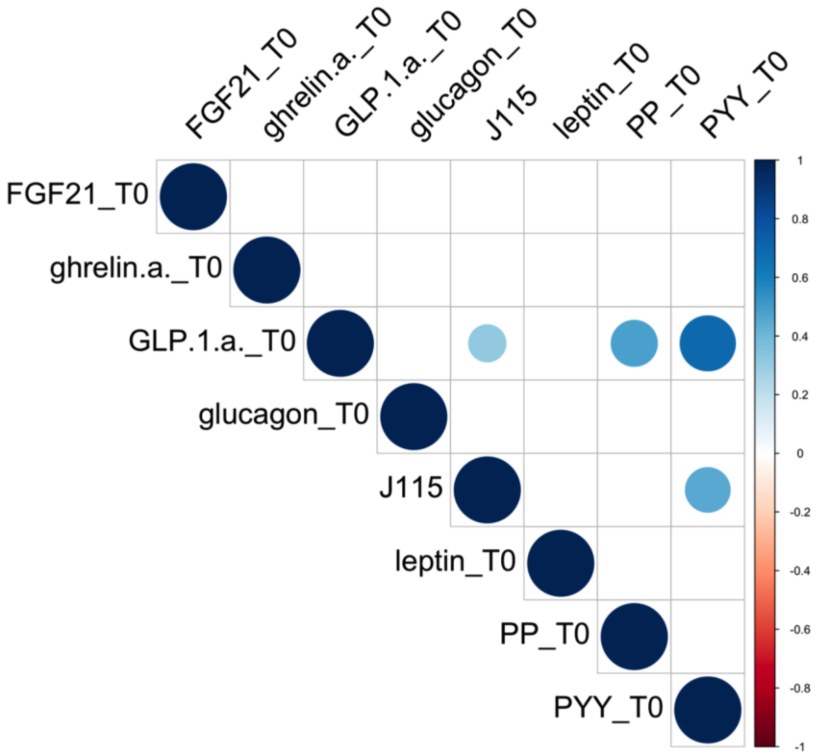

ESM Fig. 5. Correlation analysis between metabolic parameters and *D. welbionis* (J115) measured in the blood of a subset of fasted subjects (n=49) included in the cohort prior intervention (baseline, M0)

The presence of a circle indicates that the correlation is significant, $p < 0.05$ (Spearman's correlation test)**.** The circle size relates with the absolute value of correlation. The bigger the circle size, the higher the correlation. Blue indicates positive correlations and red negative correlations.
